# Journal Bills

**Published:** 1893-12

**Authors:** 


### JOURNAL BILLS.

We have sent a friendly letter with statement of account to nearly all who are indebted to this journal. We have done this, first, to get what is due us, because we need it, and secondly, to have everyone understand plainly how they stand with us. We have received many letters, saying: "Do not hesitate in sending us statement of account;" "it is our neglect," or "carelessness;" "we beg pardon," etc. Some say: "we thought we had paid you." A few: "we have paid you;" and occasionally one breaks out; "Stop the JOURNAL; we won't be annoyed by duns." A few, because we have allowed them to run behind, ignore us entirely. Now, we send receipt to every one that pays us, and we receipt for the year he is in arrears; so that it is an easy matter to prove the payment, should a second call for that year be made. Paste your receipt in the volume you have paid, so that it can be found. Some at the beginning of the year pay for the year past, and in time they conclude that it is for the present year. Time flies quickly and we forget. An editor can't keep accounts in his head. He aims to keep his books accurately, though mistakes are possible. The receipt will soon show up the error. Then we are ready to acknowledge and confess and beg pardon. We cannot aflord to carry a subscriber from year to year without a response to our demands, and finally, when no answer, when patience ceases to be a virtue, we draw on you through the bank and ask you to honor. This is the last call. Those in arrears four and six dollars must expect this when no response is given to the bill sent by mail.—*Exchange.*

The above is as true as it is humiliating. Why should an honest man refuse to pay for his medical journals or kick at bills long since due when presented to him?—*Ex.*

We will ask that all subscribers that are due us, will remit this month· Thanking you in advance, we wish you all a merry Christmas.

